# Facile synthesis of a WO_*x*_/Cs_*y*_WO_3_ heterostructured composite as a visible light photocatalyst†

**DOI:** 10.1039/c7ra12355h

**Published:** 2018-02-12

**Authors:** N. Tahmasebi, S. Madmoli

**Affiliations:** Department of Science, Jundi-Shapur University of Technology Dezful Iran tahmasebi@jsu.ac.ir

## Abstract

In this work, a WO_*x*_/Cs_*y*_WO_3_ heterostructured composite was synthesized *via* a simple pyrolysis method followed by heat treatment under a reducing atmosphere. Optical absorption results revealed the WO_*x*_/Cs_*y*_WO_3_ heterostructured composite exhibits a strong absorption tail in the Vis and NIR regions which could have important implications for its photoactivity. The photocatalytic performance of synthesized samples with different Cs/W molar ratios was evaluated by the photodegradation of RhB in aqueous solution under simulated solar light irradiation. The results revealed that the photocatalytic activity of the WO_*x*_/Cs_*y*_WO_3_ composite is much higher than those of pure tungsten bronze (Cs_*x*_WO_3_, *x* = 0.32, and 0.5) and pure WO_2.83_ samples, where 90% RhB was degraded after 160 min irradiation. Also, the WO_*x*_/Cs_*y*_WO_3_ composite exhibits excellent photocatalytic activity for the degradation of MO, MB, RhB, and MG aqueous solution under visible light irradiation. It is proposed that the higher photocatalytic activity of the WO_*x*_/Cs_*y*_WO_3_ composite could be attributed to the greater surface adsorption of dye molecules, intense light absorption in the visible and NIR regions, and photogenerated electron–hole separation.

## Abstract 2

In this work, a WO_*x*_/Cs_*y*_WO_3_ heterostructured composite photocatalyst was synthesized *via* a simple pyrolysis method followed by heat treatment under a reducing atmosphere.

## Graphical abstract

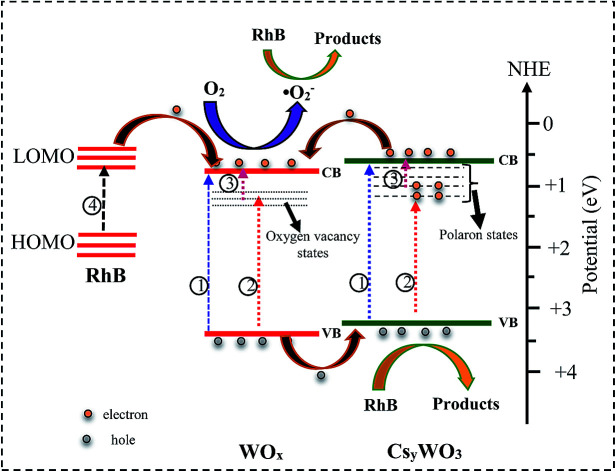

## 1 Introduction

In recent years, semiconductor photocatalysis, owing to its ability to degrade organic dyes and contamination using solar energy, has attracted significant attention as a potential means to purify wastewater.^1,2^ To date, the TiO_2_ semiconductor is the most studied photocatalyst because its wide band gap (∼3.2 eV) is suitable for photocatalytic performance under UV light irradiation.^3^ However, the ultra violet (UV) region makes up a very small part of the total content of incoming solar energy, ∼5%, while the visible (Vis) and near-infrared (NIR) regions contain around 45%, and 50% of the total incoming energy.^4^ Therefore, to avoid the huge waste of the visible part of solar light, the synthesis of visible light active photocatalysts has been highly desired. Tungsten oxide is an n-type semiconductor with a band gap between 2.4 and 2.8 eV that can degrade a wide range of organic contaminations under visible light irradiation.^5–7^ However, because of rapid electron–hole recombination rate, stoichiometric WO_3_ is often known as an insufficient photocatalyst under ambient condition.^6^ Recently, to overcome this drawback, the photocatalyst activity of sub-stoichiometric tungsten oxide compound such as WO_*x*_ (2 < *x* < 3) has widely been investigated.^8,9^ It is found that the sub-stoichiometric tungsten oxide exhibits noticeable activity in the photocatalytic degradation of dye molecules due to intense light absorption in visible and near-infrared regions and strong surface adsorption of dye molecules.^8,10,11^ Among all tungsten sub-oxide, monoclinic W_18_O_49_ has received considerable attention, and the adsorption and photocatalytic performance of other compounds rarely has been investigated.

Tungsten bronzes are a group of the non-stoichiometric compound with the general formula of M_*x*_WO_3_, (M represents an alkali metal) where *x* is in the range of 0 < *x* < 1.^12^ Tungsten bronze (M_*x*_WO_3_) nanostructures have a wide variety of application such as NIR shielding devises, gas sensors, photothermal, and photocatalyst owing to their excellent optical and electrical properties.^13–16^ The crystal structures of this compound are based on corner sharing of WO_3_ octahedra, with the metal cations located in the formed channels. Based on the nature of metal cations and its fractional occupancy in the channels, this compound can be classified into four type: perovskite tungsten bronze (PTB), tetragonal tungsten bronze (TTB), hexagonal tungsten bronze (HTB) and intergrowth tungsten bronze (ITB).^17,18^ More recently, alkali tungstate and tungsten bronze have been regarded as photocatalyst. Up to now, only a few studies have been reported on the photocatalytic degradation of the organic compound by pure alkali tungstate and tungsten bronze.^19–21^ Li *et al.* synthesized the Cs_*x*_WO_3_ nanorods that exhibited full-spectrum-response photocatalytic activity.^20^ In 2010 by Wang *et al.* the photocatalytic activity of the sodium tungstate (Na_*x*_WO_3+*x*/2_) nanowires bundles was also reported, and the intrinsic mechanism of the enhanced photocatalytic activity was described based on “band-filling mechanism” and the existence of X^5+^.^19^ In other reports, the photocatalytic activity of pure alkali tungstate and tungsten bronze is insignificant, and the coupling with other semiconductor has been applied to improve the photocatalytic performance.^22–24^ Man *et al.* prepared the Cs_*x*_WO_3_/BiOCl heterojunction which showed a higher photocatalytic activity than that of a pure Cs_*x*_WO_3_ and pure BiOCl under visible light irradiation.^24^ However, the photocatalytic performance of tungsten bronzes is still poor, compared with other semiconductor photocatalyst. Also, up to now, although the hexagonal cesium tungsten bronze crystal structure extensively has been studied,^20^ the experimental investigation of other cesium tungsten bronze crystal structures such as intergrowth tungsten bronze and cubic-pyrochlore phases rarely have been reported.^25–27^

To improve the photocatalyst performance of semiconductors, a variety of strategies have been applied to suppress rapid combination of photogenerated electrons and holes, as well as to increase the intensity of absorbing light.^28^ These include noble metal loading,^29^ hydrogen treatment^30^ and heterojunction photocatalysts.^31^ Among these, heterojunction photocatalysts have attracted most attention owing to their effectiveness for separation of photogenerated electron–hole pairs and more intense light absorption.^32–34^ Heterojunction is the interface between two coupled semiconductors with unequal band structure.^31^ It is reported that the heterojunction between different phases of same semiconductor photocatalyst is more effective for electrons–holes separation, due to the intimate contact of the two phase at atomic levels.^31^ Zhang *et al.* synthesized a metallic/semiconductor H_*x*_WO_3_/WO_3_ photocatalyst which can degrade organic dyes under visible and NIR irradiation. The NIR absorption and the improvement of photocatalytic activity were attributed to the oxygen vacancy levels below the conduction band of H_*x*_WO_3_ and electron transfer from WO_3_ to H_*x*_WO_3_, respectively.^35^ In 2015 by Cui *et al.* a WO_2_/Na_*x*_WO_3_ composite phase was prepared by a high temperature reduction process of hexagonal-Na_*x*_WO_3_ nanowires bundles. The synthesized hybrid photocatalyst showed potential for IR-driven photocatalytic water splitting.^36^ In this work, WO_*x*_/Cs_*y*_WO_3_ heterostructured composites, the pure WO_2.83_, and pure tungsten bronze (Cs_*x*_WO_3_, *x* = 0.3 and 0.50) were synthesized by a simple pyrolysis method followed by heat treatment under a reducing atmosphere (H_2_/Ar 5%). The samples were characterized by scanning electron microscopy (SEM), X-ray diffraction (XRD), and UV-vis diffuse reflectance spectra. The effect of the initial molar ratio of Cs/W on the crystal structure, morphology and photocatalytic performance was investigated. The photocatalytic activities were evaluated by photodegradation of rhodamine B (RhB) as pollutant model under light irradiation. It was found the photocatalytic activity of WO_*x*_/Cs_*y*_WO_3_ composite was highly enhanced compared to the pure phase of tungsten bronzes (Cs_*x*_WO_3_, *x* = 0.3 and 0.66) and WO_2.83_.

## 2 Experimental section

### 2.1 Synthesis of photocatalysts

All reagents used in this work were analytical grade and employed without further purification. Tungstic acid (H_2_WO_4_), hydrogen peroxide (H_2_O_2_), ammonia (NH_3_), citric acid (C_6_H_8_O_7_), cesium carbonate (Cs_2_CO_3_), polyvinylpyrrolidone, methylene blue (MB), rhodamine B (RhB), methyl orange (MO), and malachite green (MG) were purchased from Sigma-Aldrich. In a typical experimental, 0.76 g tungstic acid powder was loaded into a beaker, and then 4.5 mL hydrogen peroxide was poured into the beaker and was stirred at 50 °C for 1 h. Then, 1 mL of NH_3_ and 2 g citric acid was added to the above solution under vigorous stirring for 30 min. After that, different amount of cesium carbonate was added and followed by constant stirring for 30 min. The initial molar ratio of Cs/W was set to be 0.00, 0.10, 0.30, and 0.66. Finally, 0.02 g polyvinylpyrrolidone (PVP, *M*_w_ = 1 300 000) was added to the solution. The solution was stirred for 14 h to obtain a homogeneous sol. Then, the obtained sol was transferred into a crucible and thermal decomposition was done with a heating rate of 1 °C min^−1^ for 8 h from the room temperature in the air, and followed by heat treatment at 500 °C for 2 h. Finally, the synthesized powder samples with different Cs/W molar ratio were annealed in a hydrogen atmosphere (H_2_/Ar = 5%) at the temperature of 650 °C for 1 h with the flow of hydrogen at 10 mL h^−1^ to get the final product.

### 2.2 Characterization

Scanning electron microscopy (TESCAN VEGA model) and field emission scanning electron microscopy (TESCAN MIRA3 XMU-FESEM) were utilized to study the morphology and microstructure of the samples and to determine the chemical composition with energy dispersive X-ray spectroscopy (EDS) spectrum. X-ray diffraction (XRD) with Cu Kα radiation (*λ* = 1.54178 Å) at 40 kV was used to study the crystalline phases. The UV-vis diffuse reflectance spectra were measured by UV-vis spectrometer (Avaspec-2048-TEC model, Avantes) using BaSO_4_ as a reference in the range of 200–800 nm. The absorption spectra were obtained by kubelka–Munk method. The photoluminescence (PL) emission spectra were performed with spectrometer (Thermo Lumia model) at room temperature with the excitation wavelength of 260 nm.

### 2.3 Photocatalytic and adsorption experiments

The photocatalytic performance of the synthesized samples with different Cs/W molar ratio in the degradation of RhB was evaluated by using a 55 W xenon lamp as simulated solar light source. In a typical experiment, 60 mg photocatalyst was dispersed in 60 mL of RhB solution (25 mg L^−1^ for WO_2.83_ and WO_*x*_/Cs_*y*_WO_3_ samples, and 5 mg L^−1^ for Cs_0.3_WO_3_ and Cs_0.5_WO_3_ samples) in a 250 mL beaker reactor that was equipped with a water-cooling jacket to adjust the reaction temperature. The lamp located 10 cm away from the reaction solution. Also, the visible light activity of photocatalysts was investigated by photocatalytic degradation of an aqueous solution of RhB, MG, and MB (40 mL, 20 mg L^−1^) in the presence of 40 mg photocatalyst and MO (40 mL, 20 mg L^−1^) in the presence of 20 mg photocatalyst under a white light LED lamp (30 W). Prior to light irradiation (xenon or LED), the solution was magnetically stirred in the dark for 80 min to reach a complete adsorption–desorption equilibrium between the photocatalyst and pollutant model. During the light irradiation, at given interval, 2 mL of solution was sampled and centrifuged to remove the photocatalyst. The residual pollutant concentrations were estimated by the UV-vis spectrophotometer measurement. The photodegradation of the pollutant model solution was estimated according to the following formula: *η* = [(*C*_0_ − *C*)/*C*_0_] × 100%, where *C* and *C*_0_ are the absorbance of the pre- and post-irradiation of the RhB solution, respectively.

To compare the dye adsorption capability of the synthesized samples, 60 mg photocatalyst was dispersed in 60 mL of RhB solution (5 mg L^−1^) in the dark, and the solution was magnetically stirred. Then at given interval 2 mL of solution was collected and centrifuged, and the RhB concentration was determined by UV-vis spectroscopy.

## 3 Results and discussion

### 3.1 XRD analysis

**Fig. 1:**
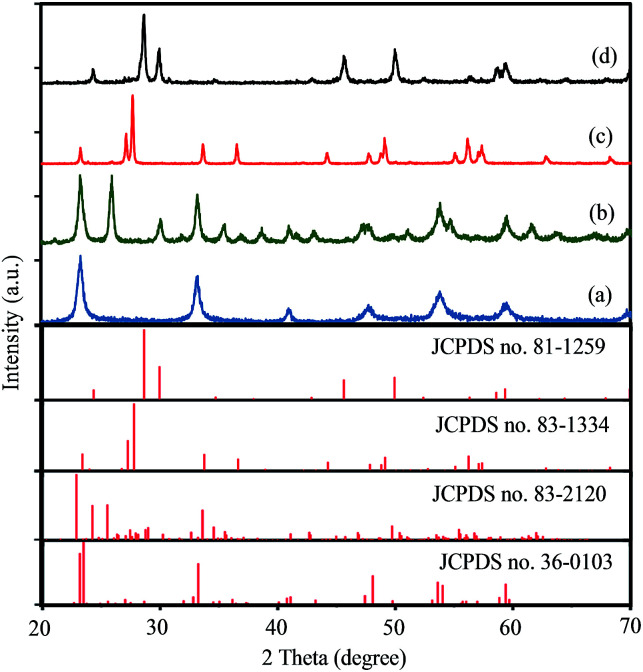
XRD patterns of synthesized samples with different cesium to tungsten molar ration (*A* = Cs/W): (a) 0.00, (b) 0.10, (c) 0.30, and (d) 0.66.

The crystal structures of products synthesized with initial Cs/W molar ratio of *A* = 0.00, 0.10, 0.30 and 0.66 were characterized by XRD pattern. As shown in Fig. 1, all the diffraction peaks of the synthesized samples with *x* = 0.00 and *x* = 0.30 could be indexed to pure monoclinic WO_2.83_ phase (W_24_O_68_, JCPDS no. 36-0103) and pure hexagonal Cs_0.32_WO_3_ phase (JCPDS no. 83-1334), respectively. Fig. 1(b) exhibits when the initial Cs/W molar ratio is 0.10, two series of diffraction peaks can be observed that attributed to the monoclinic phase of WO_2.83_ (JCPDS no. 36-0103) and orthorhombic Cs_0.069_WO_3_ phase (JCPDS no. 83-2120). Therefore, this sample shows a mixed phase of monoclinic-WO_2.83_ and orthorhombic-Cs_0.069_WO_3_. Fig. 1(d) shows the XRD pattern of the synthesized sample with *A* = 0.66. This figure clearly displays that all the diffraction peaks can be indexed to pyrochlore-type cubic Cs_0.5_WO_3_ phase (JCPDS no. 81-1259). Therefore, it can be concluded that the synthesized samples with initial *A* = Cs/W molar ratio of *A* = 0.00, 0.10, 0.30 and 0.50 are denoted as m-WO_2.83_, m-WO_*x*_/O-Cs_*y*_WO_3_, h-Cs_0.32_WO_3_ and c-Cs_0.5_WO_3_, respectively.

### 3.2 Morphological analysis

#### 3.2.1 SEM analysis

**Fig. 2:**
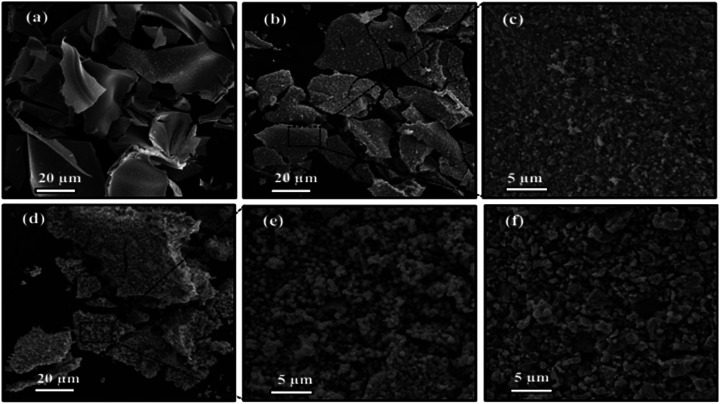
SEM images of synthesized samples with different cesium to tungsten molar ration (*x* = Cs/W): (a) *x* = 0.00, (b, c) 0.10, (d, e) 0.30, and (f) 0.66.

The morphology of samples were observed by SEM analysis. Fig. 2 shows the morphology of synthesized tungsten bronze samples with different initial cesium to tungsten molar ratio. As shown in Fig. 2(a), the sample without cesium content (m-WO_2.83_) exhibits large nanosheets with a rather smooth surface and the average size of several micrometers. The morphology of WO_2.83_/Cs_0.069_WO_3_ mixed phase has formed of a series of microsheets while a large number of nanoparticles with an average size of 200 nm have been embodied within these microsheets (Fig. 2(b and c)). The morphology of pure hexagonal tungsten bronze (Cs_0.32_WO_3_) presents only uniform nanoparticles with an average size of about 400 nm (Fig. 2(d and e)). Thus, the observed nanoparticles in the WO_2.83_/Cs_0.069_WO_3_ structure could be related to the orthorhombic tungsten bronze phase (Cs_0.06_WO_3_), so that at higher Cs/W molar ration (Cs/W > 0.1) almost all WO_3_ structure have transformed into cesium tungsten bronze nanoparticles. Furthermore, the morphology of synthesized sample with initial molar ration of Cs/W = 0.66 have irregular particles with size of 200–800 nm that have a pure cubic crystal phase (Cs_0.5_WO_3_).

#### 3.2.2 EDS analysis

**Fig. 3:**
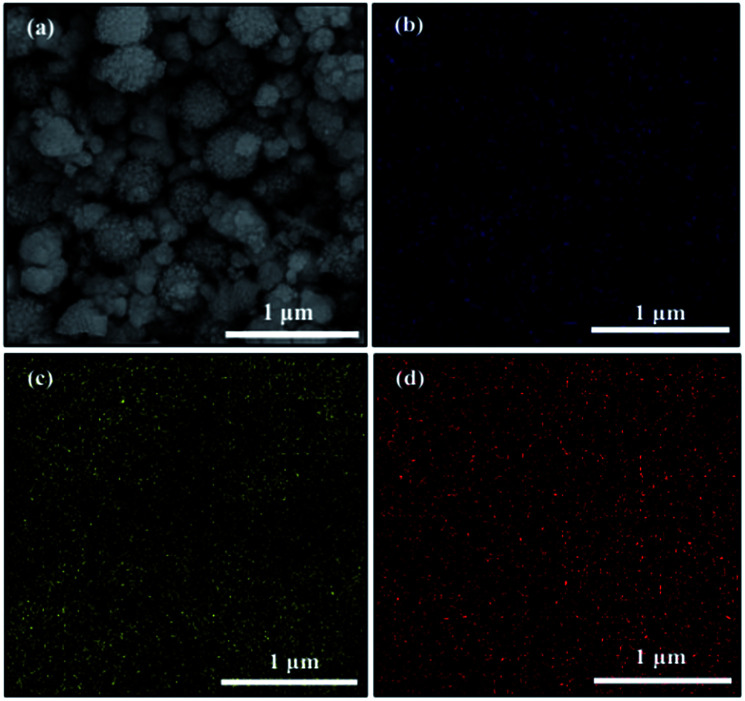
FESEM image of WO_2.83_/Cs_0.069_WO_3_ composite (a), and its corresponding elemental mappings of (b) Cs, (c) O, and (d) W elements.

The EDS analysis was used to determine the chemical composition of the synthesized samples with initial Cs/W molar ratio of *A* = 0, 0.10, 0.30 and 0.66 (Fig. S1†). This analysis revealed that the samples are composed of W, O and Cs elements, and other impurities is not detected. Also, this analysis was carried out to calculate the value of *x* in Cs_*x*_WO_3_ samples according to atomic percent of W and Cs. It is found that the calculated atomic ratio of Cs/W is equal to 0.00, 0.08, 0.31 and 0.58 for pure tungsten oxide and synthesized samples with initial Cs/W molar ration of 0.10, 0.30 and 0.66, respectively (Table 1), which are very close to the determined phases based on XRD results. Furthermore, to reveal the elemental distribution of elements, the elemental mapping is performed on synthesized sample with initial Cs/W molar ration of 0.10. This analysis reveals the highly uniform distribution of Cs and W elements on a microsheet (Fig. 3). Therefore, based on XRD and mapping results it can be expected that WO_2.83_ and Cs_*x*_WO_3_ are successfully composited and formed a WO_2.83_/Cs_*x*_WO_3_ mixed phase.

**Table 1:** The EDS analysis of synthesized samples with initial Cs/W molar ratio of *A* = 0, 0.10, 0.30 and 0.66

Initial Cs/W molar ratio	Atomic percent (%)			Estimated Cs/W atomic ratio by EDS
	OK	WL	CsL	
*x* = 0.00	67.14	32.86	—	—
*x* = 0.10	72.65	25.33	2.03	0.08
*x* = 0.30	70.35	22.62	7.03	0.31
*x* = 0.66	72.51	17.32	10.17	0.58

### 3.3 Photocatalytic and adsorption study

**Fig. 4:**
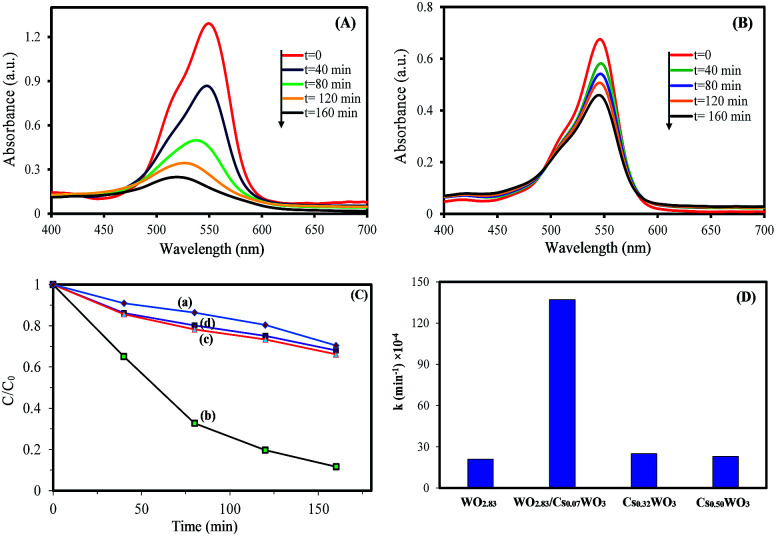
The temporal evolution of UV-vis absorption spectra of RhB solution in the presence of (A) WO_2.83_/Cs_0.07_WO_3_ composite, and (B) Cs_0.50_WO_3_ under simulated sunlight (xenon lamp), (C) photocatalytic degradation of RhB in the presence of (a) WO_2.83_, (b) WO_*x*_/Cs_*y*_WO_3_ composite, (c) Cs_0.32_WO_3_, and (d) Cs_0.50_WO_3_, and (D) photodegradation rate constant of them.

To understand the relationship between tungsten bronze crystal phase and photocatalyst performance, the photocatalytic activity of synthesized samples was evaluated by the photocatalytic decomposition of RhB in an aqueous solution as a model pollutant under simulated solar light (xenon, 55 W), and visible light (white light LED, 30 W) sources. Temporal evolution of UV-vis absorption spectra of RhB solution in the presence of WO_*x*_/Cs_*y*_WO_3_ composite and Cs_0.5_WO_3_ photocatalysts under simulated sunlight are displayed in Fig. 4(A and B). In this figure, the decrease in the absorption peak at ∼550 nm, accompanied by the exposure time, can be related to the removal of the RhB molecules in the solution. Fig. 4(C) shows the photocatalyst efficiencies of the samples under simulated solar light, where *C* and *C*_0_ were the absorption of RhB at the wavelength of 550 nm and the absorption after the adsorption equilibrium on the photocatalyst before irradiation. This figure displays that only 14%, 16% and 18% RhB can be degraded by pure WO_2.83_, Cs_0.32_WO_3_ and Cs_0.5_WO_3_, respectively, while WO_*x*_/Cs_*y*_WO_3_ composite sample with a phase junction could degraded 90% RhB after 160 min irradiation. Furthermore, the plot of ln(*C*_0_/*C*) *versus* irradiation time (*t*) displays a linear relation (Fig. S2†). Thus, the photodegradation reaction follows a pseudo-first order kinetic reaction; ln(*C*_0_/*C*) = *kt*, where *C* and *C*_0_ are the RhB concentration at initial and after *t* min irradiation, respectively, and *k* is the RhB photodegradation rate constant. The reaction rate constants were presented in Fig. 4(D). As shown in this figure, the photocatalytic activity of WO_2.83_/Cs_0.07_WO_3_, with *k* = 0.05 cm^−1^, is much higher than those of other samples.

**Fig. 5:**
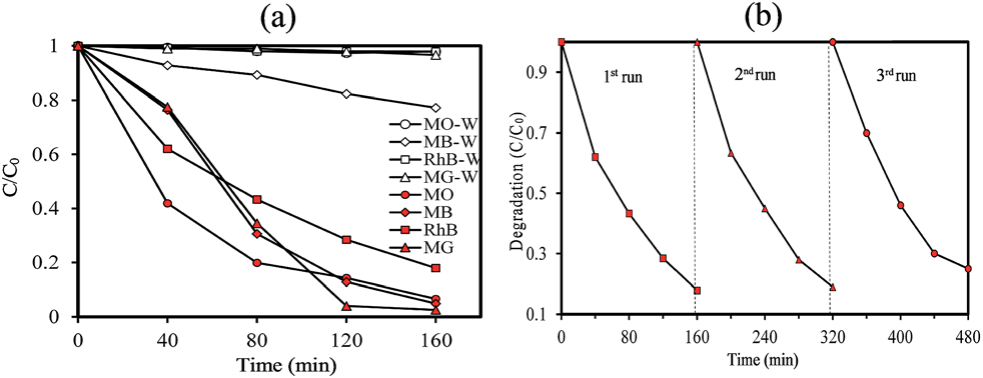
(a) The photocatalytic degradation of MG, RhB, MB, and MO solution (20 mg L^−1^) in the absence and in the presence of WO_*x*_/Cs_*y*_WO_3_ composite, and (b) recycle experiment in the photocatalytic degradation of RhB in the presence of WO_*x*_/Cs_*y*_WO_3_ composite under visible light irradiation (LED 30 W).

Then, the visible-light driven photocatalytic activity of WO_*x*_/Cs_*y*_WO_3_ composite was evaluated by photocatalytic degradation of several types of dyes. Fig. S3† shows the absorption spectra of MO, MB, RhB, and MG solution (20 mg L^−1^) over time after light irradiation with a white light LED (30 W) used as a visible light source in the presence of WO_*x*_/Cs_*y*_WO_3_ composite as the photocatalyst. As shown in Fig. 5(b), the photodegradation efficiencies of MO, MB, RhB, and MG molecules in the presence of photocatalyst after 160 min irradiation were 96%, 95%, 83%, and 98%, respectively. In contrast, the photodegradation of MO, RhB, and MG solution in the absence of any photocatalyst under visible light illumination was insignificance, and 23% of MB will be degraded under same condition. These results confirmed the good visible light activity of the composite phase for photocatalytic degradation of various dyes. Furthermore, the SEM images of the WO_*x*_/Cs_*y*_WO_3_ composite photocatalyst before and after photocatalytic reaction to photodegrade RhB molecules (under visible light irradiation) were employed to confirm its stability (Fig. S4†). The results confirm that not significant changes occurred after the photocatalytic reactions. Moreover, the WO_*x*_/Cs_*y*_WO_3_ composite photocatalyst exhibits good stability for photodegradation of RhB under visible light irradiation after three cycles (Fig. 5(b)).

**Fig. 6:**
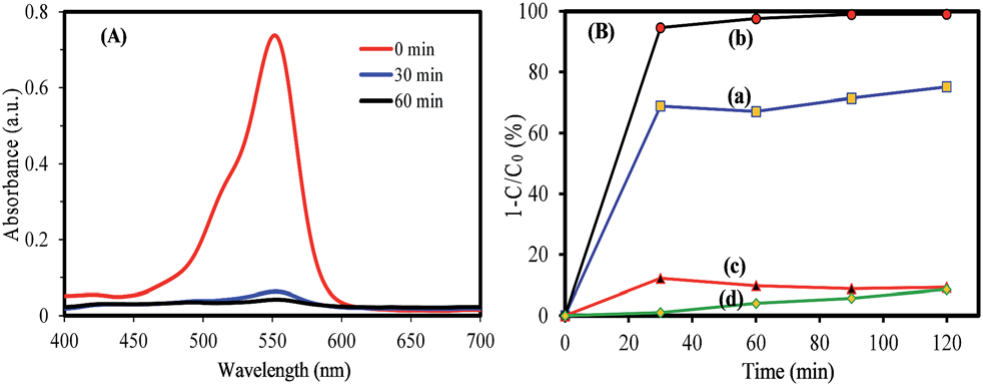
(A) UV-visible absorption spectra of a solution of RhB (50 mL, 5 mg L^−1^) in the presence of WO_*x*_/Cs_*y*_WO_3_ composite, and (B) adsorption percent of RhB on the (a) WO_2.83_, (b) WO_*x*_/Cs_*y*_WO_3_ composite, (c) Cs_0.32_WO_3_, and (d) Cs_0.50_WO_3_.

It is well known that the important factors on photocatalytic activity of a semiconductor are dye adsorption ability, light absorption for the electron–hole generation, and electron–hole separation. To study the influence of the dye adsorption ability on the photocatalyst activity of synthesized samples, the adsorption capacity of RhB on synthesized samples was investigated. Firstly, 50 mg of samples were added into 50 mL of RhB solution (5 mg L^−1^), then the solution was magnetically stirred in the dark for 80 min to reach a complete adsorption–desorption equilibrium between the photocatalyst and RhB. Fig. 6(A) shows the change of UV-vis absorption spectra of RhB solution in the presence of WO_2.83_/Cs_0.07_WO_3_ composite during adsorption. It can be seen that the intensity of absorption peak decreases with increasing the adsorption time under dark condition. Fig. 6(B) presents the evolution of the bronze samples as a function of adsorption time. This figure clearly exhibits that almost 98% RhB were removed by WO_*x*_/Cs_*y*_WO_3_ within 30 min in dark condition. This value is much higher that the adsorption capacity of WO_2.83_ (∼75%). In addition, the Cs_*x*_WO_3_ samples with *x* = 0.32 and 0.50 exhibit weak adsorption capacity (∼8% and 7%, respectively). Therefore, WO_*x*_/Cs_*y*_WO_3_ and pure WO_2.83_ sample have a much stronger dye adsorption capacity, while other Cs_*x*_WO_3_ samples (*x* = 0.32 and 0.50) have a weak adsorption capacity. To measure the specific surface area of the samples, the N_2_ adsorption–desorption curves were performed. The results are shown in Fig. S5 and Table S1.† This analysis indicated that the specific surface area of all samples is similar and insignificant (less than 4 m^2^ g^−1^). Thus, the rapid RhB adsorption of WO_*x*_/Cs_*y*_WO_3_ (∼100%) and WO_2.83_ (∼75%) in comparison to other Cs_*x*_WO_3_ samples (<8%) (*x* = 0.3 and 0.66) may be attributed to the presence of surface oxygen vacancies and the strong electrostatic attraction.^37,38^

**Fig. 7:**
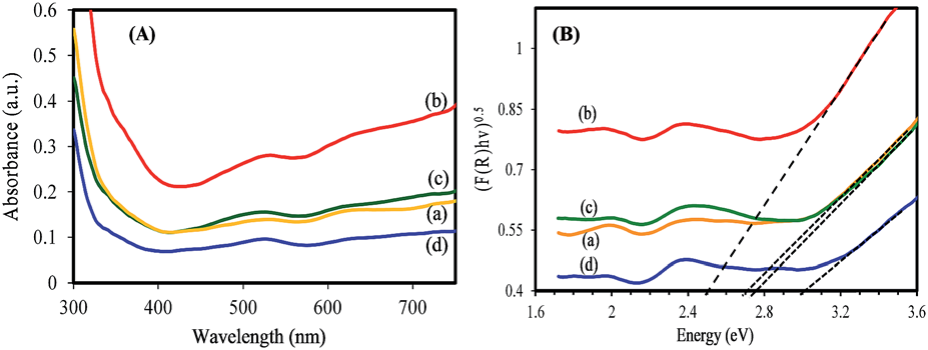
(A) UV-vis absorption spectra (converted from DRS spectra) and (B) the plot of (*F*(*R*)*hv*)^1/2^*vs. hv* of (a) WO_2.83_, (b) WO_*x*_/Cs_*y*_WO_3_ composite, (c) Cs_0.32_WO_3_, and (d) Cs_0.50_WO_3_ samples.

The light absorption for electron–hole generation is another key factor that has a significant effect on photocatalysis performance of a semiconductor. Therefore, the optical properties of synthesized samples have been investigated. The synthesized samples exhibit a blue color that the color gradually changes to a dark blue color (Cs_0.50_WO_3_) with increasing Cs/W molar ratio (Fig. S6†). The strong blue color could be related to the intense light absorption ability that is useful to create electron–hole pairs.^39^Fig. 7(A) displays the UV-vis absorption spectra of WO_2.83_, pure tungsten bronze samples (Cs_*x*_WO_3_: *x* = 0.32, and 0.50), and WO_*x*_/Cs_*y*_WO_3_ composite. It is observed that in addition to the absorption in UV region, all samples exhibit strong absorption in the visible region. Furthermore, one can observe the WO_*x*_/Cs_*y*_WO_3_ composite sample exhibits more intense light absorption that can be attributed to the strong interaction between WO_2.83_ and Cs_0.069_WO_3_ phases. The optical band gap of the samples is obtained from the equation: (*F*(*R*)*hν*)^*n*^ = *B*(*E* − *E*_g_), where *F*(*R*) is the kubelka–Munk function, *hν* is the photon energy, *B* is a constant and *n* is 2 or 1/2 for direct or indirect transition, respectively. The *E*_g_ value is determined by extrapolating the linear region of (*Ahν*)^1/2^ plot to *hν* = 0. It is found that the energy gap of Cs_*y*_WO_3_ pure phase shows a blue shift with increasing *x* value. Fig. 7(B) indicates that the energy gap of pure WO_2.83_, Cs_0.32_WO_3_ and Cs_0.5_WO_3_ phases are approximately 2.70 eV, 2.75 and 3.00 eV, respectively. However, the band gap value of WO_*x*_/Cs_*y*_WO_3_ composite phase is estimated to be 2.50 eV. The relatively low-value band gap of composite phase compared to pure phases can be attributed to the coupling of WO_2.83_ and Cs_0.069_WO_3_ phases and strong interaction between them.^32,40^

**Fig. 8:**
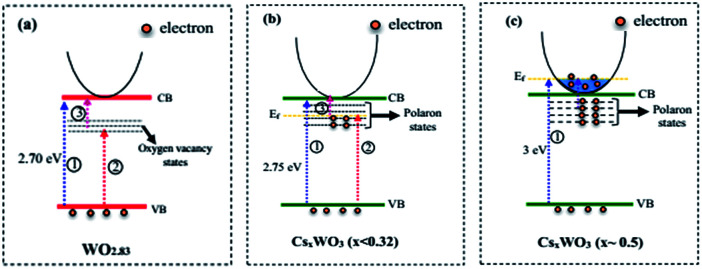
Schematic representation of band structure of (a) WO_2.83_, (b) WO_2.83_/Cs_*x*_WO_3_ (*x* < 0.32), and (c) Cs_*x*_WO_3_ samples (*x* ∼ 0.5).

It is well known that the oxygen vacancies in sub-stoichiometric tungsten oxide can produce unoccupied localized states within the forbidden gap (Fig. 8(a)) that act as electron acceptor.^9,11^ On the other hand, when Cs molecules are doped into WO_3_ lattice these molecules are dissociated as Cs ions and electrons (Cs, Cs^+^ + e^−^). The Cs ions are localized in the tungsten oxide structural tunnels and weakly bounded to the WO_6_ octahedral. Also, the induced electrons are trapped in the unoccupied d-orbital of the W^6+^ and reduced it to W^5+^, and also the lattice will be distorted by repelling and attracting the surrounding negative (O^2−^) and positive (W^6+^) ions.^13,41^ The lattice distortion can produce a series of localized states below the conduction band (CB) as polaron states (Fig. 8(b and c)). It is expected that initially, the donated electrons from Cs atoms occupied the polaron states below the conduction band (Fig. 8(b)), and as Cs in the structure increases, based on band-filling mechanism some of the states within the CB are then filled, (Fig. 8(c), process 1).^19^ As a result, the band gap energy of highly doped tungsten bronze (Cs_0.5_WO_3_) increases compared with the other samples (Fig. 7(B)). According to the above mentioned explains, as illustrated in Fig. 8, the Vis and NIR light absorption (*λ* < 1100 nm) of Cs_*x*_WO_3_ (or WO_2.83_) is suggested to be due to electron transition from VB to the polaron states (or localized states) (process 2) and from polaron state (or localized states) to the CB (process 3), or by electron transition between neighbor W^5+^ and W^6+^ sites.^4,41,42^ Besides, the induced electrons can also be inserted as free electrons in the conduction band and absorbed the long wavelength of NIR (*λ* > 1100 nm) by LSPR of free electrons.^42^

**Fig. 9:**
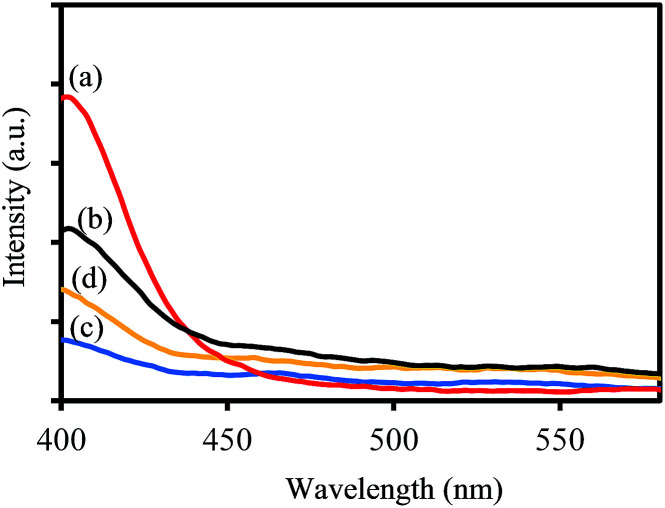
PL spectra of (a) WO_2.83_, (b) WO_*x*_/Cs_*y*_WO_3_ composite, (c) Cs_0.32_WO_3_, and (d) Cs_0.50_WO_3_ samples.

To investigate the charge separation ability of synthesized samples, the photoluminescence (PL) emission spectra were recorded at room temperature. As shown in Fig. 9, the PL spectra of all samples display that no PL signal could be observed in the wavelength range of 450–550 nm, demonstrating that no photogenerated electron–hole recombination occurred during the photocatalytic degradation process under visible light irradiation. These results confirm that tungsten bronze is helpful to enhance the charge separation process, which may be attributed to the polarons and oxygen vacancies states within the band gap.^43^

**Fig. 10:**
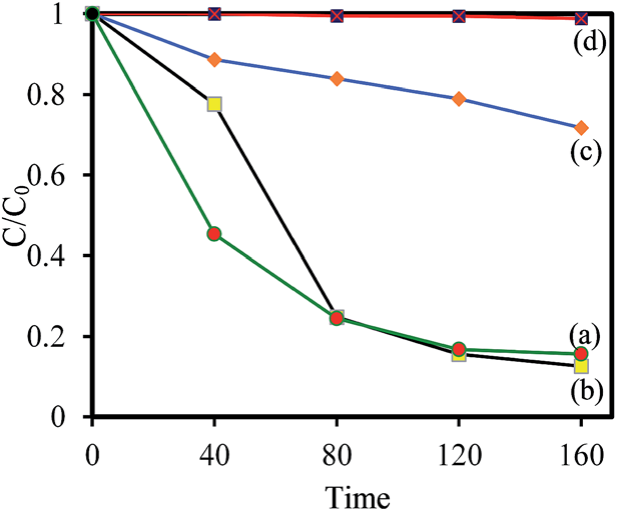
Effects of different scavengers: (a) no scavenger, (b) TBA, (c) K_2_Cr_2_O_7_, and (d) EDTA-2Na on the photocatalytic degradation of RhB by WO_*x*_/Cs_*x*_WO_3_ composite photocatalyst under Xe light irradiation.

To describe the mechanism of enhanced photocatalytic activity of WO_*x*_/Cs_*y*_WO_3_ composite the radical-trapping experiments and relative band positions were carried out. In this study, ethylene diamine tetra acetic acid disodium (EDTA-2Na), K_2_Cr_2_O_7_, and *tert*-butyl alcohol (TBA) were employed as scavengers of h^+^, e^−^, and hydroxyl radical (˙OH), respectively. As shown in Fig. 10, when EDTA-2Na selected as the scavenger, the photodegradation of RhB significantly decreases, while in the presence of K_2_Cr_2_O_7_ as scavenger the photocatalytic degradation of RhB slightly decreased. Moreover, when TBA is added as hole scavenger the photocatalytic degradation performance of RhB by WO_*x*_/Cs_*y*_WO_3_ is not affected, revealing ˙OH radicals don't take part in the photocatalytic degradation of RhB. These results suggest that although both the photogenerated electrons and holes take part in the photocatalytic reaction, the holes (h^+^) plays the important role in the degradation of RhB over WO_*x*_/Cs_*y*_WO_3_ semiconductor photocatalyst.

Furthermore, the valance band (VB) and the conduction band (CB) position of WO_*x*_ and Cs_*y*_WO_3_ are calculated using the following two equations.^44,45^

1 *E*_CB_ = *X* − *E*_e_ − 0.5*E*_g_

**Fig. 11:**
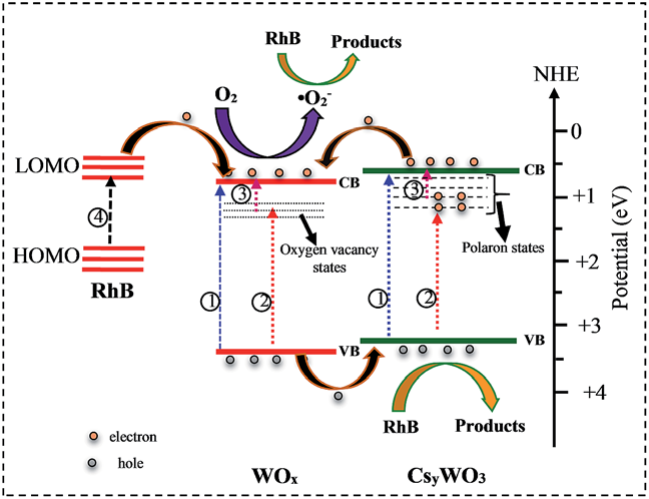
A schematic sketch of the photocatalysis mechanism of WO_*x*_/Cs_*y*_WO_3_ composite.

2 *E*_VB_ = *E*_CB_ + *E*_g_

where *E*_CB_ and *E*_VB_ are the CB and VB edge potential, respectively, *X* is the absolute electronegativity of the semiconductor, *E*_e_ is the energy of free electrons *vs.* hydrogen (4.5 eV), and *E*_g_ is the band gap energy of semiconductor. The *X* values for WO_2.83_ and Cs_0.069_WO_3_ are 6.53 eV and 6.41 eV, respectively. Based on the DRS results, the band gap of WO_2.83_ and Cs_0.069_WO_3_ are considered as ∼2.70 eV and ∼2.75 eV, respectively. Therefore, the bottom of the CB and the top of the VB of the WO_2.83_ are 0.68 eV and 3.38 eV, respectively, and the CB and VB of the Cs_0.069_WO_3_ are estimated to be 0.56 eV and 3.26 eV, respectively. Therefore, as shown in Fig. 11, the WO_*x*_/Cs_*y*_WO_3_ composite exhibits a type-2 heterostructure.^31^ As a result, the photogenerated electrons in the CB of Cs_0.07_WO_3_ can be transferred to the CB of WO_*x*_, and inversely the photogenerated holes in the VB of WO_2.83_ are transferred to the VB of Cs_*y*_WO_3_. Thus, the photogenerated electrons and holes are effectively separated, which is beneficial to suppress electron–hole recombination, and as a result can significantly enhance the photocatalytic activity. On the other hand, the RhB molecules can also be excited to form RhB* under visible light irradiation (Fig. 11, process 4). Then, the adsorbed RhB* molecules on the photocatalyst surface could transfer electrons to the CB of WO_2.83_, and react with the adsorbed O_2_ molecules to generate ˙O_2_ radicals, which would be considered as part of photodegradation activity under visible light irradiation.

## 4 Conclusions

In summary, we fabricated pure WO_2.83_, pure cesium tungsten bronze (Cs_*x*_WO_3_*x* = 0.32 and 0.50) and a WO_*x*_/Cs_*y*_WO_3_ heterostructured composite by a simple pyrolysis method. The crystal structure and microstructure of the samples were investigated by XRD and SEM analysis. The photocatalytic activity of synthesized samples was evaluated by the photodegradation of RhB in an aqueous solution as a model pollutant. The results revealed that the monoclinic/orthorhombic WO_2.83_/Cs_0.069_WO_3_ composite shows higher photocatalytic activity compared to the pure WO_2.83_ and pure tungsten bronze phase (Cs_*x*_WO_3_, *x* = 0.32 and 0.50). Moreover, the WO_*x*_/Cs_*y*_WO_3_ composite displays excellent photocatalytic activation under visible light irradiation, the photodegradation efficiencies of MO, MB, RhB, and MG molecules in the presence of photocatalyst after 160 min visible light irradiation (white light LED lamp) were 96%, 95%, 83%, and 98%, respectively. The enhanced photocatalytic performance of WO_*x*_/Cs_*y*_WO_3_ composite sample is attributed to the strong dye adsorption capability, intense light absorption, and effective separation of electron–hole pairs which could be attributed to the surface oxygen vacancies, polaron states below the conduction band, and the strong interaction between WO_2.83_ and Cs_0.069_WO_3_ phases, respectively.

## Conflicts of interest

There are no conflicts to declare.

## Supplementary Material

RA-008-C7RA12355H-s001
