# Supplementary material for: Facile synthesis of a WOx/CsyWO3 heterostructured composite as a visible light photocatalyst
Source: RSC Adv. 2018 Feb 12;8(13):7014–21. doi: 10.1039/c7ra12355h (PMC9078393; doi:10.1039/c7ra12355h)
Supplement: RA-008-C7RA12355H-s001 [file RA-008-C7RA12355H-s001.pdf]

Electronic Supplementary Material (ESI) for RSC Advances.
This journal is © The Royal Society of Chemistry 2018

SUPPORTING INFORMATION

Facile synthesis of WO_x/ Cs_yWO_3 heterostructured composite as a visible light photocatalyst

N. Tahmasebi[a]*, S. Madmoli[a]

[a]Jundi-Shapur University of technology, Dezful, Iran

*Correspondence to: tahmasebi@jsu.ac.ir

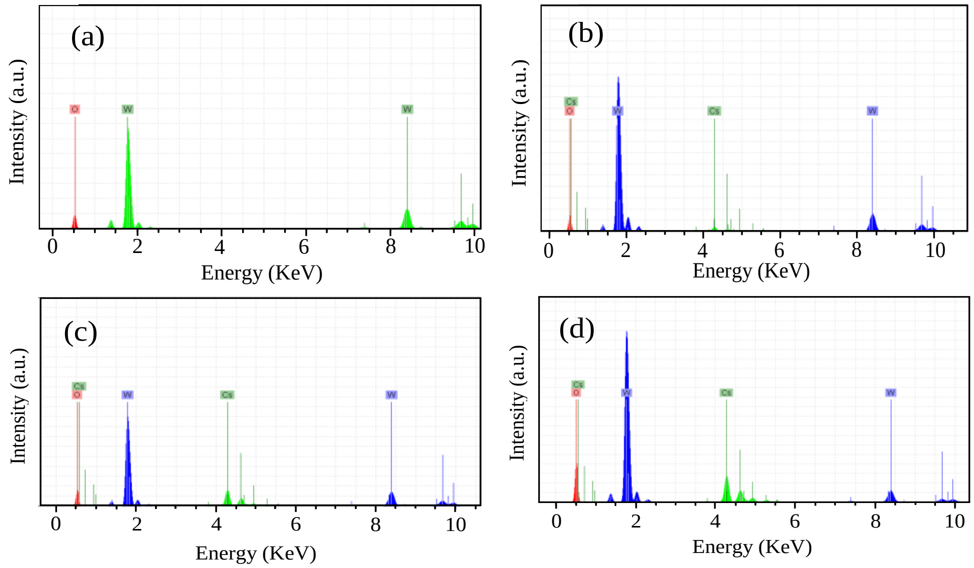

Fig. S1 EDS patterns of synthesized samples with initial cesium to tungsten molar ration (A=Cs/W) of (a) A=0.00, (b) 0.10, (c) 0.30, and (d) 0.66.

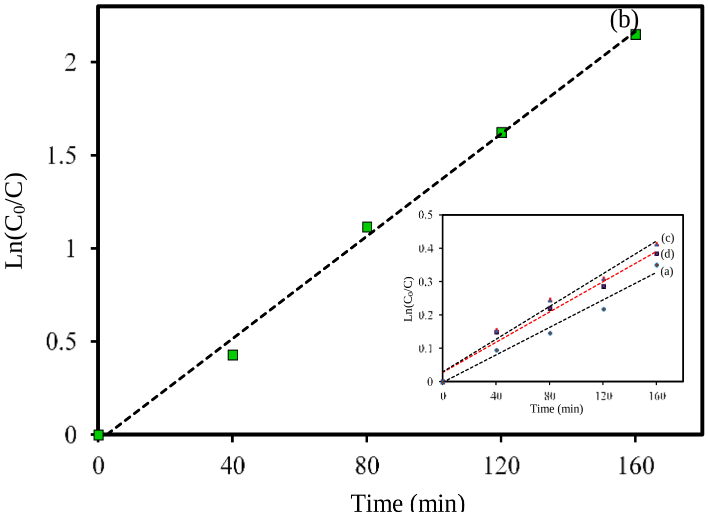

Fig. S2. Photodegradation rate constant of synthesized samples with initial cesium to tungsten molar ration (A=Cs/W) of (a) A=0.00, (b) 0.10, (c) 0.30, and (d) 0.66.

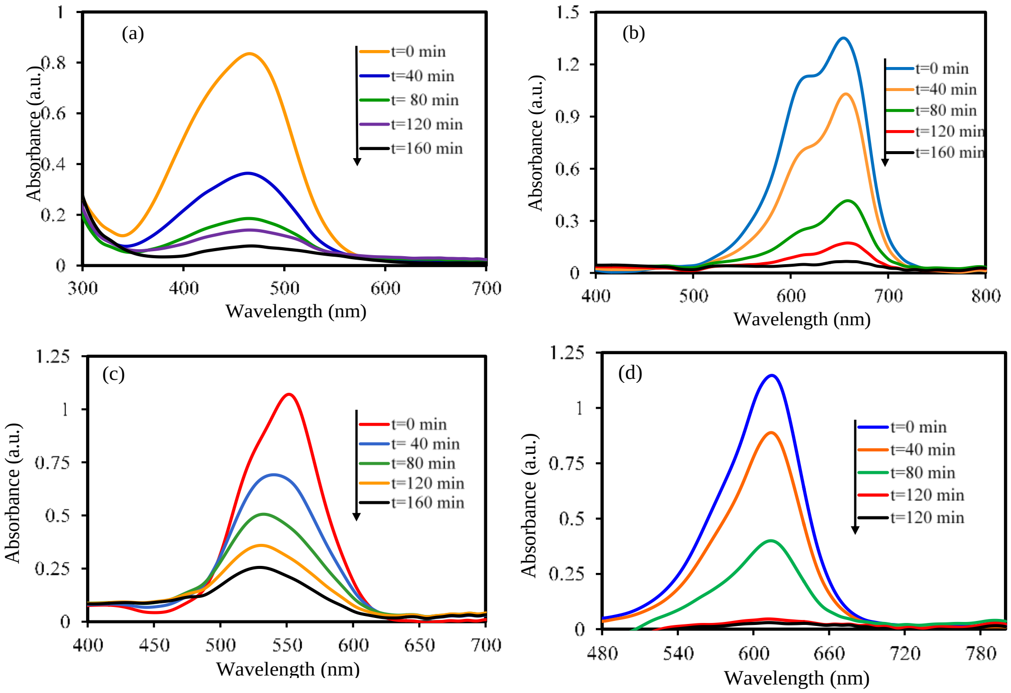

Fig. S3 The temporal evolution of UV-Vis absorption spectra of (a) MO, (b) MB, (c) RhB, and (d) MG in the presence of WO_x/Cs_yWO_3 composite under visible light (LED 30 W) irradiation.

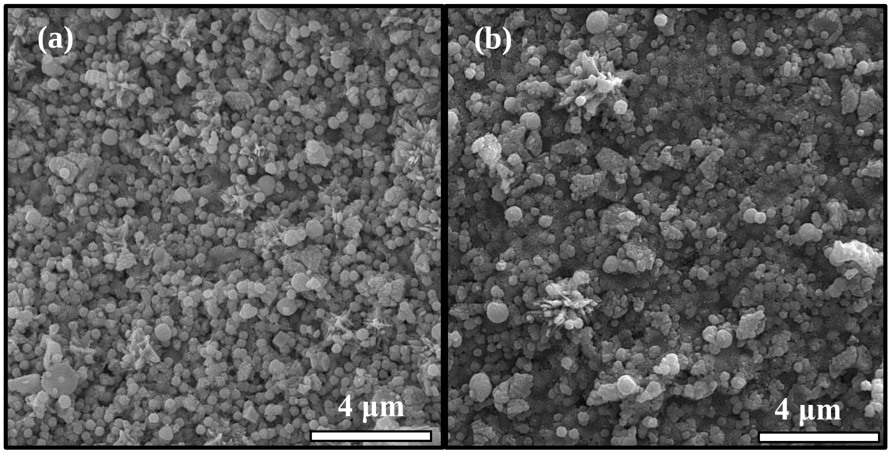

Fig. S4 SEM image of WO_x/Cs_yWO_3 composite (a) before and (b) after photocatalytic degradation of RhB solution under visible light irradiation.

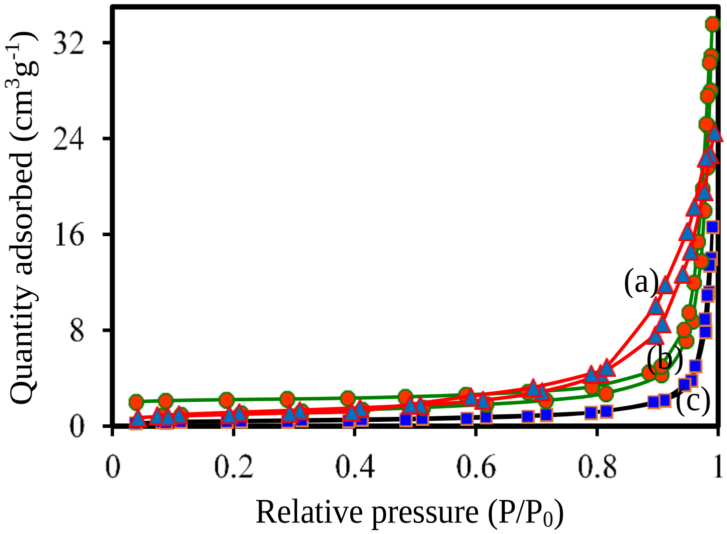

Fig. 5 Nitrogen adsorption-desorption isotherm curves of (a) $WO_{2.83}$, (b) WO_x/Cs_yWO_3, and (c) $Cs_{0.3}WO_3$.

Table S1. BET surface area, pore volume and pore diameter of different samples

Sample	BET Surface area (m^2g^{-1})	Total pore volume (cm^3g^{-1})	Mean pore diameter (nm)
$WO_{2.83}$	3.99	0.036	36.47
WO_x/Cs_yWO_3	3.75	0.051	54.95
$Cs_{0.3}WO_3$	1.53	0.025	66.65

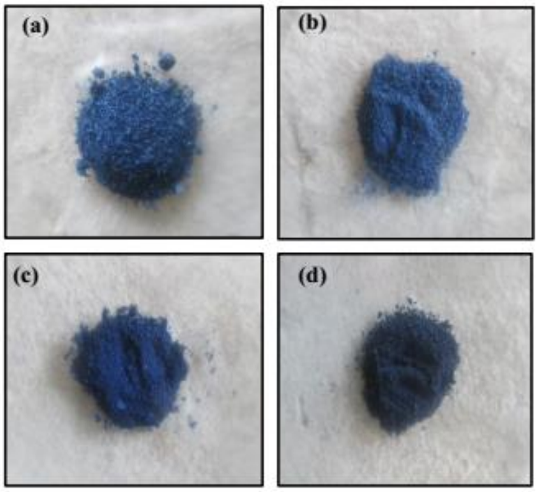

Fig. S6 The photograph image of (a) $WO_{2.83}$, (b) WO_x/Cs_yWO_3 composite, (c) $Cs_{0.32}WO_3$, and (d) $Cs_{0.50}WO_3$ samples.
